# Polycystin-1 regulates ARHGAP35-dependent centrosomal RhoA activation and ROCK signaling

**DOI:** 10.1172/jci.insight.135385

**Published:** 2020-08-20

**Authors:** Andrew J. Streets, Philipp P. Prosseda, Albert C.M. Ong

**Affiliations:** Kidney Genetics Group, Academic Nephrology Unit, Department of Infection, Immunity and Cardiovascular Disease, University of Sheffield Medical School, Sheffield, United Kingdom.

**Keywords:** Genetics, Nephrology, Genetic diseases

## Abstract

Mutations in *PKD1* (encoding for polycystin-1 [PC1]) are found in 80%–85% of patients with autosomal dominant polycystic kidney disease (ADPKD). We tested the hypothesis that changes in actin dynamics result from *PKD1* mutations through dysregulation of compartmentalized centrosomal RhoA signaling mediated by specific RhoGAP (ARHGAP) proteins resulting in the complex cellular cystic phenotype. Initial studies revealed that the actin cytoskeleton was highly disorganized in cystic cells derived from patients with *PKD1* and was associated with an increase in total and centrosomal active RhoA and ROCK signaling. Using cilia length as a phenotypic readout for centrosomal RhoA activity, we identified ARHGAP5, -29, and -35 as essential regulators of ciliation in normal human renal tubular cells. Importantly, a specific decrease in centrosomal ARHGAP35 was observed in *PKD1*-null cells using a centrosome-targeted proximity ligation assay and by dual immunofluorescence labeling. Finally, the ROCK inhibitor hydroxyfasudil reduced cyst expansion in both human *PKD1* 3D cyst assays and an inducible *Pkd1* mouse model. In summary, we report a potentially novel interaction between PC1 and ARHGAP35 in the regulation of centrosomal RhoA activation and ROCK signaling. Targeting the RhoA/ROCK pathway inhibited cyst formation in vitro and in vivo, indicating its relevance to ADPKD pathogenesis and for developing new therapies to inhibit cyst initiation.

## Abstract 2

Polycystin-1, the major protein mutated in autosomal dominant polycystic kidney disease, activates centrosomal RhoA activity via interaction with the Rho-GAP protein ARHGAP35, resulting in shorter cilia.

## Graphical abstract

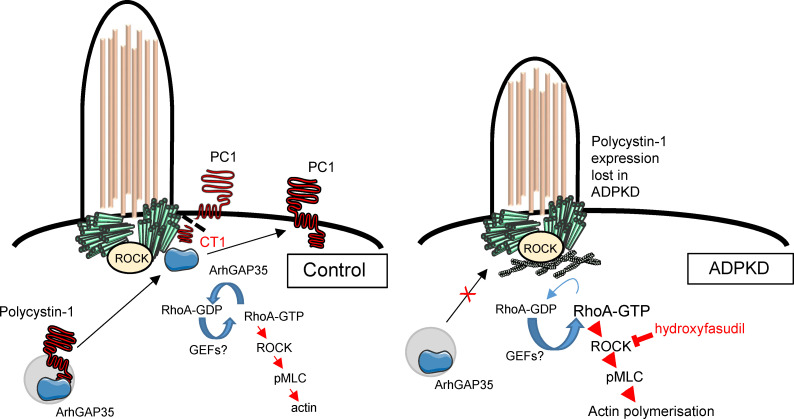

## Introduction

Autosomal dominant polycystic kidney disease (ADPKD) is the most common inherited cause of end-stage kidney failure in humans (1). It has an estimated clinical prevalence of less than 1 in 2000, although its genetic prevalence could be higher due to many asymptomatic undiagnosed cases in the general population (2). Around 10% of patients on renal replacement therapy are due to ADPKD, making it a disease of considerable personal, societal, and economic impact. At present, only 1 drug, tolvaptan, has been approved for use in humans to slow disease progression (3).

Mutations in 2 genes, *PKD1* and *PKD2*, account for the majority of cases (>90%) with ADPKD. In mutation-negative patients, a combination of mosaicism in *PKD1* or mutations in other cystic genes (*DNAJB11, GANAB*) have been detected, although these still account for a small percentage of such cases (4, 5). *PKD1* encoding PC1 is the causative gene in 80%–85% of patients, making this the major ADPKD gene. It has also been recognized that other rarer cystic genes may exert their effects through alterations in the expression, processing, or localization of PC1, making an understanding of PC1 function central to the understanding and treatment of most forms of human PKD (6). However, this has been a challenging task given the size, complexity, and posttranslational modifications of the protein (7).

The cellular phenotype of ADPKD is well described but is highly complex with alterations in many pathways reported. These include changes in proliferation, apoptosis, cell-cell and cell-matrix adhesion, differentiation, apicobasal polarity, fluid secretion, cilia function, directional migration, and matrix deposition (7). In the majority of studies, it has been difficult to assign a specific phenotype to PC1 function. We tested the hypothesis that *PKD1* mutation leads to significant changes in actin cytoskeleton dynamics giving rise to several features of the cystic phenotype. In this study, we report that dysregulation of compartmentalized centrosomal RhoA signaling mediated by a specific RhoGAP (ARHGAP35) leads to increased Rho kinase (ROCK) activation in *PKD1* mutant cells.

## Results

### Changes in actin organization are a striking feature of the ADPKD cellular phenotype.

**Figure 1:**
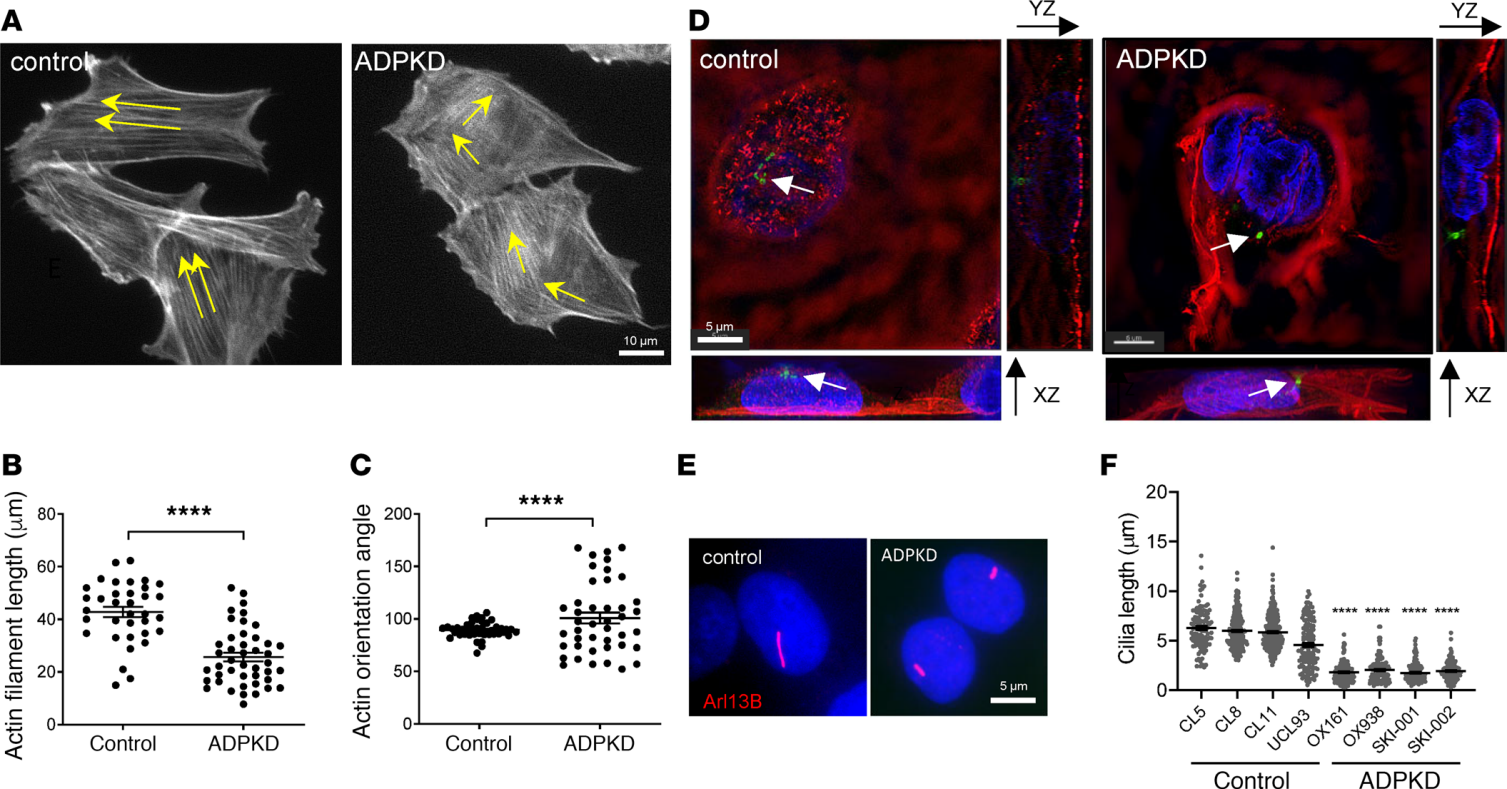
The ADPKD cellular phenotype is associated with structural changes in actin organization and reduced cilia length. (**A**) Phalloidin labeled F-actin showing a more disorganized actin cytoskeleton in *PKD1* cystic cells (OX161) compared with control (UCL93) cells. (**B** and **C**) The length of actin filaments was significantly reduced and their normal parallel orientation more variable in *PKD1* compared with control cells (N = 60 cells; significance determined by 2-tailed Student’s *t* test). (**D**) 3D-SIM confocal images of phalloidin-stained cells. Actin fibers can be seen predominantly orientated to the base of control UCL93 cells. In contrast, actin fibers were thicker and frequently localized to the apical surface of OX161 cells. Increased stress fibers were also present. Cilia labeled with Arl13b (green, arrows) are shorter. (**E**) Primary cilia were visualized in quiescent control and ADPKD cell lines after serum starvation by immunofluorescence labeling of Arl13b (red) and nuclei (blue). (**F**) Cilia length was significantly reduced in a panel of human *PKD1* cystic compared with noncystic cell lines (*n* = 8 patient-derived cell lines, *N* = 250 cells counted; significance determined by 1-way ANOVA corrected (Tukey) for multiple comparison. ****P < 0.0001. ADPKD, autosomal dominant polycystic kidney disease; SIM, structured illumination.

In preliminary studies, we observed that abnormalities in the actin cytoskeleton were a common feature of patient-derived *PKD1* cystic cell lines. Actin fibers appeared to be thicker, shorter, and more disorganized in *PKD1* cells compared with noncystic controls (Figure 1, A–C). 3D structured illumination (3D-SIM) confocal images of phalloidin-stained cells confirmed the general increase in cortical F-actin at the apical and basolateral surface, stress fiber formation, and reduced cell height in a *PKD1* cell line (OX161) compared with a noncystic control (UCL93) (Figure 1D and Supplemental Videos 1 and 2; supplemental material available online with this article; https://doi.org/10.1172/jci.insight.135385DS1); in control cells, actin fibers were predominantly seen at the basal surface. In addition, we made the surprising observation that, under conditions promoting optimal cilia formation (48-hour serum starvation), the percentage of ciliated cells as well as cilia length were reduced in *PKD1* cystic cells (Figure 1, E and F, and Supplemental Figure 1A). These changes were not related to prolonged in vitro culture or immortalization since a significant reduction in the cilia length and percentage of ciliated cells were also detected in nephrectomy tissue obtained from 2 patients with *PKD1* (OX161 and SKI-001) compared with noncystic patients (Supplemental Figure 1, B and D). Similar changes were confirmed in cystic kidney tissue from a *Pkd1* mouse model (8) (Supplemental Figure 1, C and E), excluding the possibility of secondary changes in late-stage or end-stage disease in human tissues.

### The cilia cellular phenotype is associated with increased actin polymerization and PC1 deficiency.

**Figure 2:**
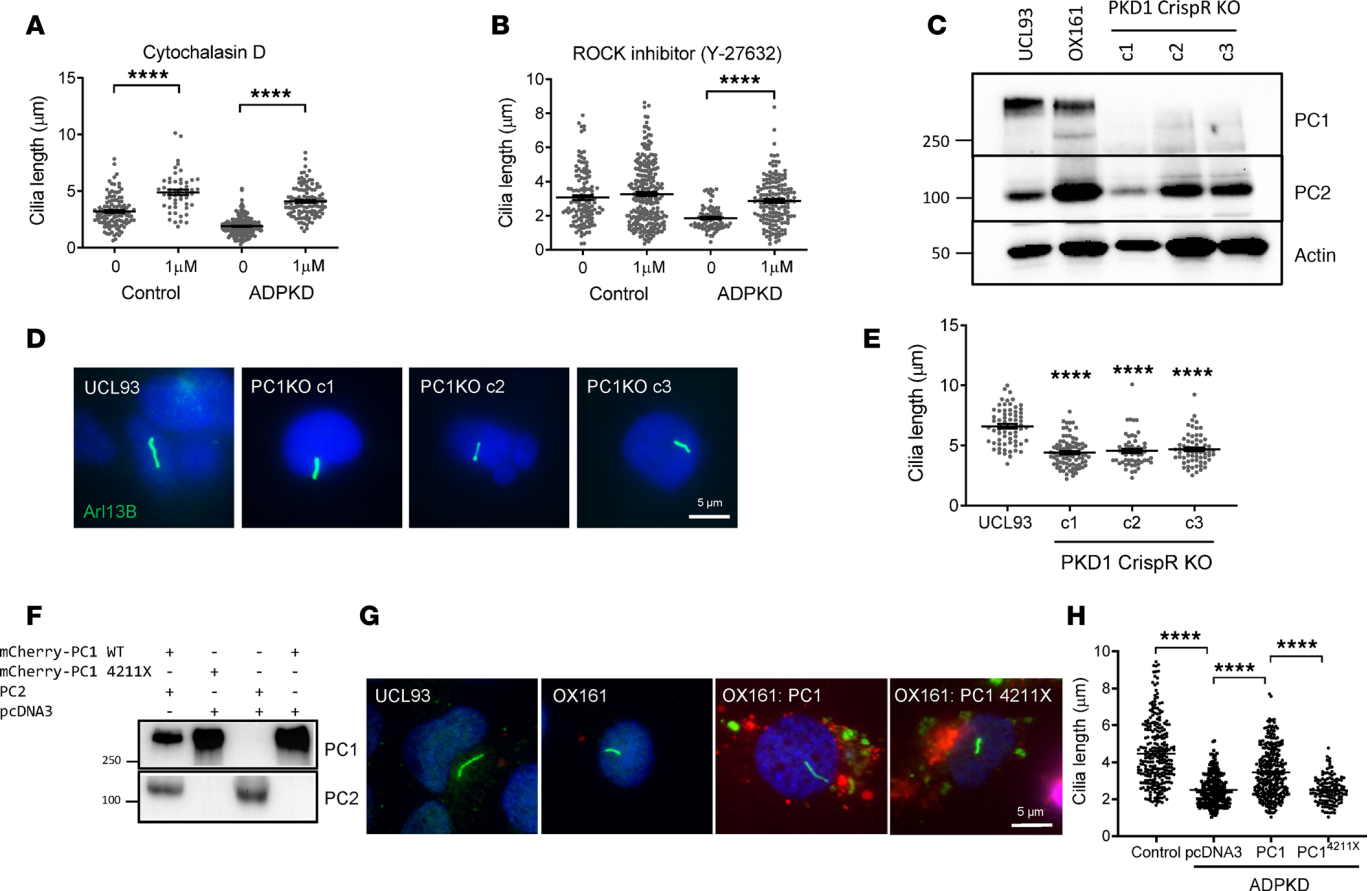
Primary cilia length correlates with PC1 expression, actin polymerization, and ROCK activity. (**A**) Cytochalasin D (1 μM, 4 hours) was associated with a significant increase in cilia length in both control (UCL93) and ADPKD (OX161) lines (*n* = 4 independent experiments, *N* = 115 cells). (**B**) The ROCK inhibitor Y-27632 (1 μM, 4 hours) rescued the cilia length defect in the ADPKD (OX161) line but had no effect on cilia length in control (UCL93) cells (*n* = 4 independent experiments, *N* = 264 cells). (**C**) Isogenic *PKD1*-null cells were generated by CRISPR/Cas9 in the parental control line, UCL93. PC1-null clones (c1–3) were expanded for study. (**D**) Primary cilia were visualized in quiescent control (UCL93) and *PKD1* null lines (PC1KO) after serum starvation by immunofluorescence labeling of Arl13b (green) and nuclei (blue). (**E**) Cilia length was significantly reduced in *PKD1* null lines (PC1KO) compared with control (UCL93) cells (*n* = 3 independent experiments, *N* = 67 cells). (**F**) Expression of mCherry-PC1, mCherry-PC1-4211X, or CFP-PC2 in transfected HEK293 cells showing bands of the expected size by immunoblotting for PC1 (7e12) or PC2 (G20). (**G**) Representative images of primary cilia in UCL93 control cells and OX161 cystic cells showing partial rescue of cilia length (Arl13b, green) in cells cotransfected with mCherry-PC1 (red) and CFP-PC2 but not mCherry-PC1-4211X (red) and CFP-PC2. (**H**) Expression of mCherry-PC1 was associated with a significant increase in cilia length compared with mCherry-PC1 4211X or pcDNA3 transfected control OX161 cells (*n* = 3 independent experiments, *N* = 264 cells). ****P < 0.0001. Significance determined by 2-tailed Student’s *t* test (**A** and **B**). Significance determined by 1-way ANOVA corrected (Dunnett) for multiple comparison (**E** and **H**). PC1, polycystin-1; ADPKD, autosomal dominant polycystic kidney disease.

In view of the observed structural defects in actin organization, we hypothesized that increased actin polymerization could be the major underlying defect leading to shorter cilia in *PKD1* cystic cells. Although the primary cilium is a microtubule-based organelle, it has been recognized that apical actin filaments are necessary to stabilize cilia formation by promoting centrosome migration, basal body docking, and axoneme growth (9). These changes have been reported in rare ciliopathies such as Bardet-Biedel and Meckel syndromes (10, 11), although the role of actin in ciliogenesis in ADPKD has not been previously investigated. Strikingly, actin depolymerization induced by cytochalasin D (1 μM) increased cilia length in both normal and *PKD1* cystic cells to a similar degree (Figure 2A), linking the degree of actin polymerization to cilia stabilization. In contrast, the ROCK inhibitor Y-27632 restored cilia length in *PKD1* cells to that found in normal cells but had no effect on cilia length in normal cells (Figure 2B), implicating a mechanistic link between PC1 deficiency, RhoA activation, and increased ROCK activity.

To confirm that this was the case, we generated *PKD1-*null cells from the parental normal control human tubular cell line (UCL93) by CRISPR/Cas9 mutagenesis. Three isogenic clones were selected for further study based on the absence of PC1 expression (Figure 2C). In early passage cells, we again observed a clear reduction in cilia length (Figure 2, D and E), confirming a direct link to PC1 deficiency. In agreement with this, cotransfection of WT mCherry-PC1 (with WT PC2) into OX161 cells resulted in a partial rescue of the cilia phenotype; in contrast, no rescue was observed in cystic cells expressing a mutant mCherry-PC1-4211X truncation deleting most of the C-terminus (Figure 2, F–H). To investigate if PC2 was similarly implicated in this pathway, we generated *PKD2-*null cells in the same line using the same strategy. *PKD2*-null cells, however, had normal cilia lengths compared with the parental line (UCL93), indicating that the cilia phenotype is specific to PC1 and/or that PC2 plays a permissive but nonessential role (Supplemental Figure 1F).

### RhoA activation is increased in PKD1 mutant or null cells and kidney tissues.

**Figure 3:**
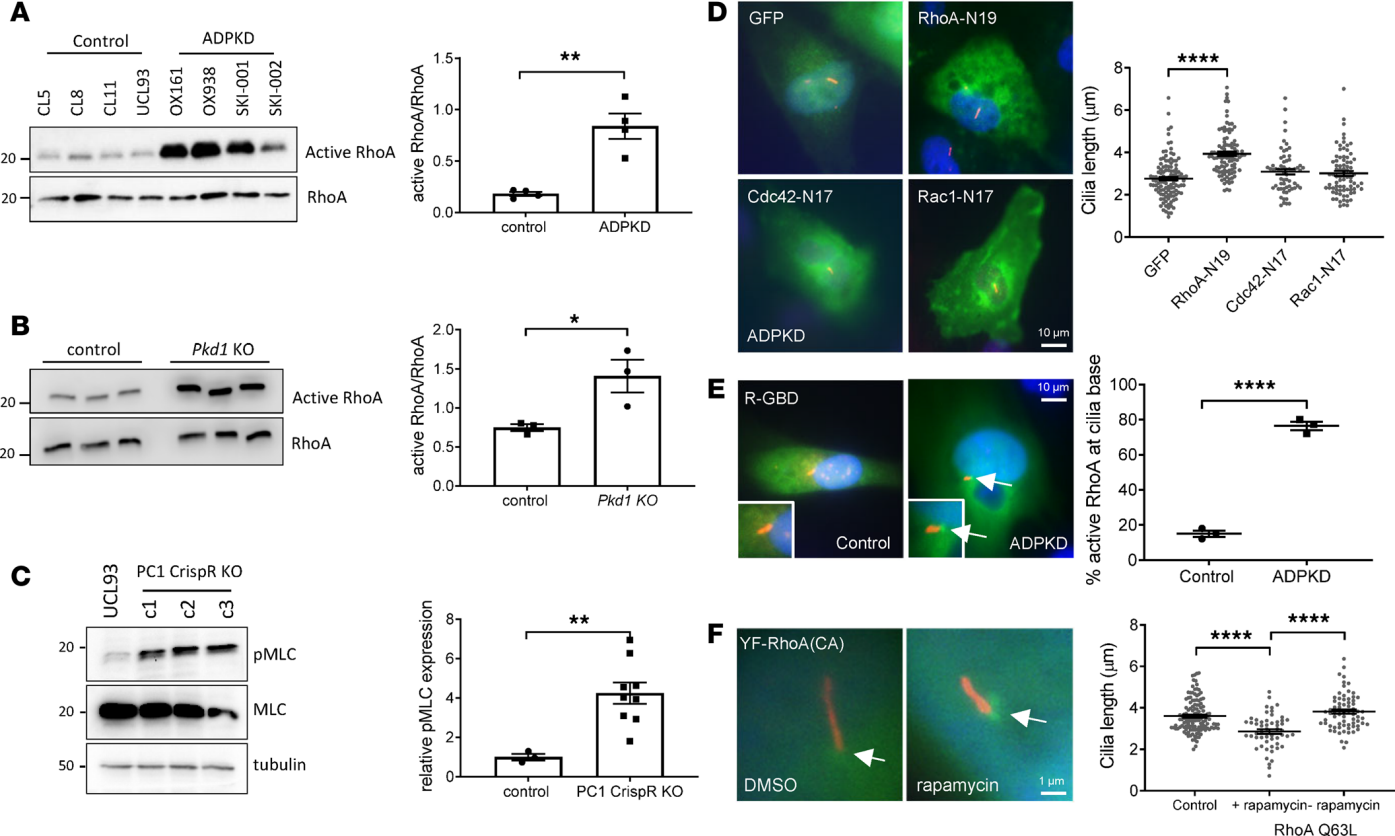
Total and centrosomal RhoA and ROCK activity is increased in ADPKD models and alters cilia length. (**A**) GTP-RhoA was significantly increased in *PKD1* cystic cell lines compared with control cells (*n* = 4) using a Rhotekin-GTP pulldown assay. (**B**) GTP-RhoA was significantly increased in *Pkd1-*knockout kidneys compared with controls (*n* = 3). (**C**) Phosphorylation of myosin light chain (pMLC), a major downstream target of ROCK, was significantly upregulated in isogenic *PKD1*-null cells (*n* = 3). (**D**) Expression of dominant negative RhoA (T19N) in *PKD1* cells resulted in a significant increase in cilia length compared with dominant negative Cdc42 (N17) or Rac1 (N17) (*n* = 3 independent experiments, *N* = 81 cells). (**E**) Active RhoA was localized using a GTP-RhoA biosensor (R-GBD) in control and *PKD1* cells. In ciliated cells, active RhoA (GFP) was visualized at the cilia base (arrows) and was significantly increased in *PKD1* cells (*n* = 3 independent experiments, *N* = 22 cells). Insets show cilia under higher magnification (original magnification, ×1000). (**F**) Rapamycin-inducible centrosomal targeted expression (arrow) of constitutively active RhoA (Q63L) was associated with a significant decrease in cilia length in control cells compared with uninduced cells (*n* = 3 independent experiments, *N* = 70 cells) *P < 0.05, **P < 0.01, ****P < 0.0001. Significance determined by 2-tailed Student’s *t* test (**A–C** and **E**). Significance determined by 1-way ANOVA corrected (Dunnett) for multiple comparison (**D** and **F**). ADPKD, autosomal dominant polycystic kidney disease.

To explore the potential mechanistic link between increased ROCK activity and PC1 deficiency, the activation of RhoA in disease cells and tissues was measured using a Rhotekin-RBD pulldown assay designed to recognize GTP-RhoA. A significant increase in total GTP-RhoA was detectable in *PKD1* cystic cells compared with controls (Figure 3A) and confirmed in cystic kidneys of *Pkd1* mice (Figure 3B). Under conditions to promote cilia formation, we also observed a significant increase in total GTP-Cdc42 but not of GTP-Rac1 in *PKD1* cystic cells (Supplemental Figure 2, A–C). We also detected a significant increase in the phosphorylation of myosin light chain (MLC), a major downstream effector of ROCK that activates actin contraction and stabilization, in *PKD1*-null cells (Figure 3C).

### Centrosomal RhoA activation is increased in PKD1 cells and directly regulates cilia length.

To exclude an additional role for Cdc42 or Rac1 in regulating cilia length, we next expressed dominant negative (DN) versions of each GTPase in *PKD1* cystic cells. As indicated, expression of DN RhoA (N19) but not DN Cdc42 (N17) or DN Rac1 (N17) increased cilia length in OX161 cells (Figure 3D). We hypothesized that apart from the total cellular increase in active GTP-RhoA, there was likely to be an increase in active RhoA at the centrosome or basal body compartment to account for the cilia phenotype in *PKD1* cells. Using a fluorescent active RhoA biosensor (12), we directly visualized significantly increased active RhoA in this compartment in *PKD1* cells compared with controls (Figure 3E). To confirm that an increase in active centrosome RhoA can result directly in shorter cilia, we exploited a rapamycin-inducible system to express constitutively active RhoA (Q63L) at the centrosome in control cells (Supplemental Figure 3B). As shown, this resulted in significantly shorter cilia compared with noninduced cells (Figure 3F).

### Knockdown of several centrosomal ARHGAPs decreased cilia length in control cells.

**Figure 4:**
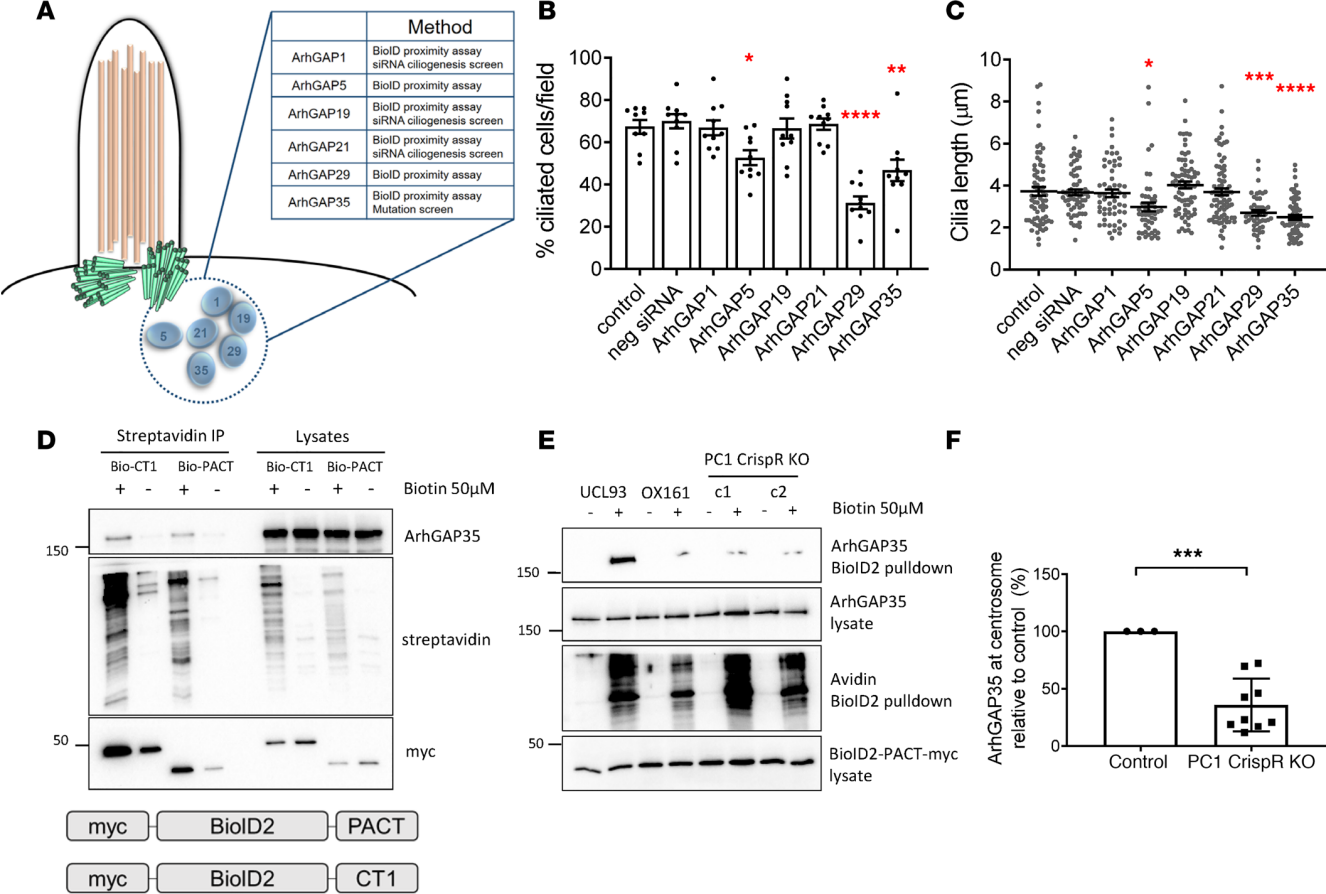
Centrosomal ARHGAP35 localization is regulated by PC1. (**A**) Candidate centrosomal ARHGAP proteins identified from cilia/centrosome databases and siRNA cilia screens. Six potential ARHGAPs were reported in at least 2 studies. (**B** and **C**) SiRNA knockdown in control cells (UCL93) demonstrated that reduced ARHGAP5, -29, and -35 expression resulted in a reduction of the percentage of ciliated cells and cilia length, whereas knockdown of ARHGAP1, -19, and -21 was neutral for ciliogenesis (*n* = 3 independent experiments, *N* = 50 cells). (**D**) Centrosomal expression of endogenous ARHGAP35 in HEK293 cells was demonstrated using a proximity ligation assay with a myc-tagged BioID2-PACT fusion protein (BioID2-PACT), which localizes to centrosomes. In addition, endogenous ARHGAP35 was labeled by a second myc-tagged BioID2 fusion protein containing the C-terminus of PC1 (BioID2-CT1), indicating that both proteins are likely interaction partners. (**E** and **F**) There was reduced centrosomal expression of ARHGAP35 in *PKD1* cystic (OX161) or null (c1, c2) cells compared with control (UCL93) cells in the proximity ligation assay using BioID-PACT (*n* = 3). *P < 0.05, **P < 0.01, ***P < 0.001, ****P < 0.0001. Significance determined by 2-tailed Student’s *t* test (**F**). Significance determined by 1-way ANOVA corrected (Dunnett) for multiple comparison (**B** and **C)**. PC1, polycystin-1.

The regulation of RhoA activity between the active GTP-bound state and the inactive GDP-bound state is mediated by ARHGEFs, which promote GTP binding and ARHGAPs, which promote GTP hydrolysis (13). The increase in active RhoA at centrosomes which we observed in *PKD1* cells led us to hypothesize that a possible explanation could be loss of specific centrosomal ARHGAPs due to PC1 deficiency. Database and literature mining of several studies of the cilia/centrosome proteome and siRNA ciliogenesis screens identified 6 likely centrosomal ARHGAP proteins reported in at least 2 studies (Figure 4A) (14–20).

We next conducted a focused siRNA screen on these 6 candidate ARHGAPs (ARHGAP1, -5, -19, -21, -29, -35) in control cells (UCL93), achieving knockdown of greater than 80% by qPCR (Supplemental Figure 3A). Of note, knockdown of ARHGAP5, -29, and -35 but not ARHGAP1, -19, and -21 was found to significantly reduce ciliogenesis (Figure 4, B and C).

### Centrosomal ARHGAP35 localization is reduced in PC1-null cells.

The centrosomal localization of ARHGAP5, -29, and -35 in control UCL93 cells was experimentally confirmed using a BioID2 proximity ligation assay (PLA) linked to the centrosomal targeting PACT domain (Figure 4D and Supplemental Figure 3, C and D). Of interest, all 3 proteins were also detected using a second BioID2 PLA linked to the PC1 C-terminus (CT1), suggesting their close proximity to PC1 at centrosome and/or noncentrosome compartments (Figure 4D and Supplemental Figure 3D).

**Figure 5:**
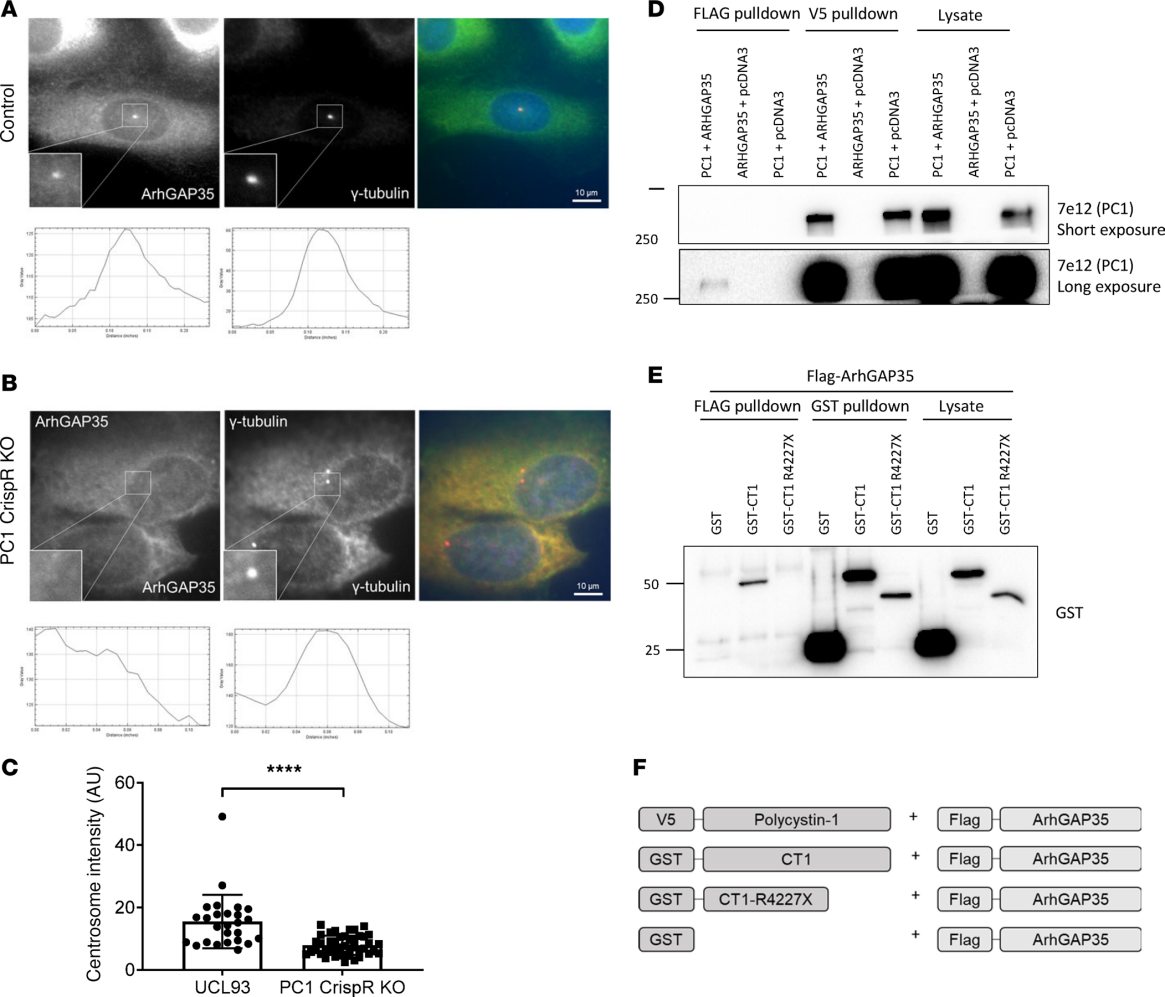
Centrosomal ARHGAP35 interacts with PC1. (**A–C**) Centrosomal ARHGAP35 expression was visualized by colocalization with γ-tubulin and labeling with a specific ARHGAP35 antibody: a clear reduction in centrosomal labeling was observed in *PKD1-*null cells compared with controls. Representative images showing ARHGAP35 and γ-tubulin staining in control (**A**) and *PKD1-*null cells (**B**). Staining intensity in the defined area of the centrosome was quantified by ImageJ (**C)** in 3 independent *PKD1* null clones (c1–3) compared with control cells (UCL93) (*n* = 3 independent experiments, *N* = 51 cells). (**D**) Coexpressed full-length FLAG-ARHGAP35 and V5-PC1 proteins coimmunoprecipitate in HEK293 cells indicate their likely interaction. (**E**) GST-pulldown of FLAG-ARHGAP35 with GST-CT1 but not GST-CT1-R4227X indicates the PC1 C-terminus as the likely interaction domain. Representative blots from 3 independent experiments. ****P < 0.0001. Significance determined by 2-tailed Student’s *t* test. (**F**) Schematic diagram of the expression constructs used for PC1 and ARHGAP35 experiments. PC1, polycystin-1.

To test whether the localization of any of these centrosomal ARHGAPs could be regulated by PC1, we compared their centrosomal localization in *PKD1* mutant (OX161) and *PKD1-*null cells with isogenic control cells (UCL93) using the PACT-targeted BioID2 PLA. In the absence of PC1, a striking decrease in centrosome localization of ARHGAP35 was noted (Figure 4, E and F). We confirmed the reduction in centrosomal ARHGAP35 in *PKD1-*null cells by dual immunofluorescence, with specific antibodies to γ-tubulin and ARHGAP35 (Figure 5, A–C). In contrast, no significant change in centrosomal ARHGAP5 and ARHGAP29 was observed in the absence of PC1 (Supplemental Figure 3E).

Co-IP of full-length epitope-tagged ARHGAP35 and PC1 expressed in HEK293 cells confirmed their direct interaction (Figure 5D). ARHGAP35 was shown to bind the PC1 C-terminus since it did not bind to a truncated PC1 mutant protein (CT1-R4227X) in GST-pulldown assays (Figure 5E).

### ROCK inhibition inhibits cyst growth in vitro and in vivo.

**Figure 6:**
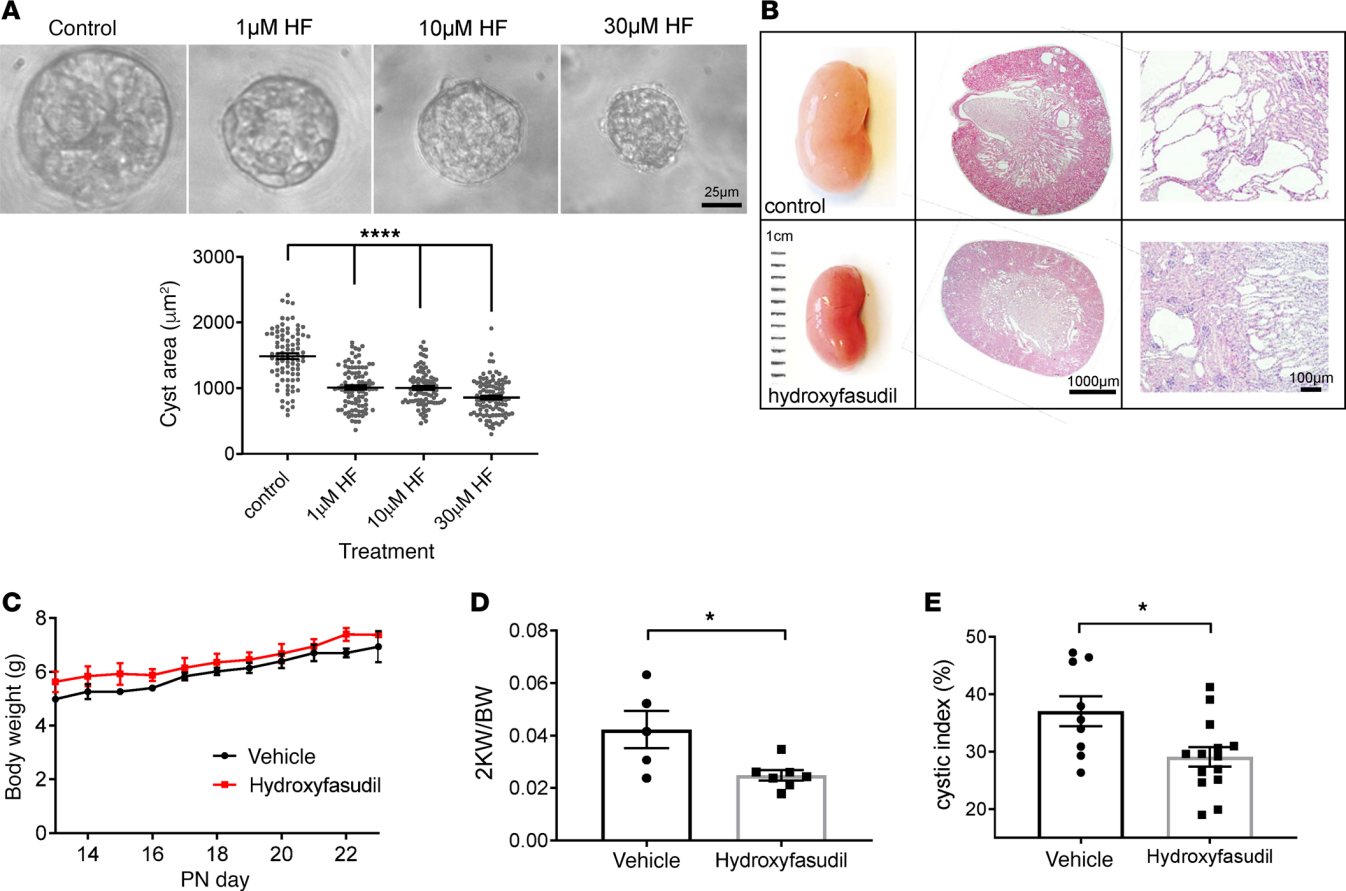
ROCK inhibition reduces cyst growth in vitro and in vivo. (**A**) The ROCK inhibitor hydroxyfasudil reduced cyst growth of a patient-derived *PKD1* cystic line (OX161) in 3D cyst assays. Representative images of cysts after 12 days treatment. Average cyst area was reduced at all concentrations (1, 10, 30 μM) tested (*n* = 3 independent experiments, *N* = 96 cysts). (**B**) Hydroxyfasudil-treated *Pkd1* mice (10 mg/kg/day by i.p. injections from PN16) (*n* = 7) showed similar weight gain to vehicle (dH_2_0)-injected control animals before (*n* = 5) and after treatment for 7 days (PN22). Significance determined by 1-way ANOVA test. (**C**–**E**) Hydroxyfasudil treatment was associated with a reduction in kidney size, fractional weight (2KW/BW), and cyst formation (cystic index). *P < 0.05, ****P < 0.0001. Significance determined by 2-tailed Student’s *t* test (**D** and **E**). Significance determined by 1-way ANOVA corrected (Dunnett) for multiple comparison (**A**).

The increase in total and centrosomal GTP–bound RhoA in *PKD1*-deficient cells, the normalization of cilia length by a ROCK inhibitor, and the increased expression of pMLC led us to conclude that ROCK might be a relevant therapeutic target in PKD1. We first tested the efficacy of a second ROCK inhibitor, hydroxyfasudil, in 3D cyst assays using a patient-derived *PKD1* cystic line (OX161). Hydroxyfasudil (1–30 μM, 7 days) was associated with a significant decrease in cyst area, confirming a likely link between the increased ROCK activity and cyst growth (Figure 6A).

**Figure 7:**
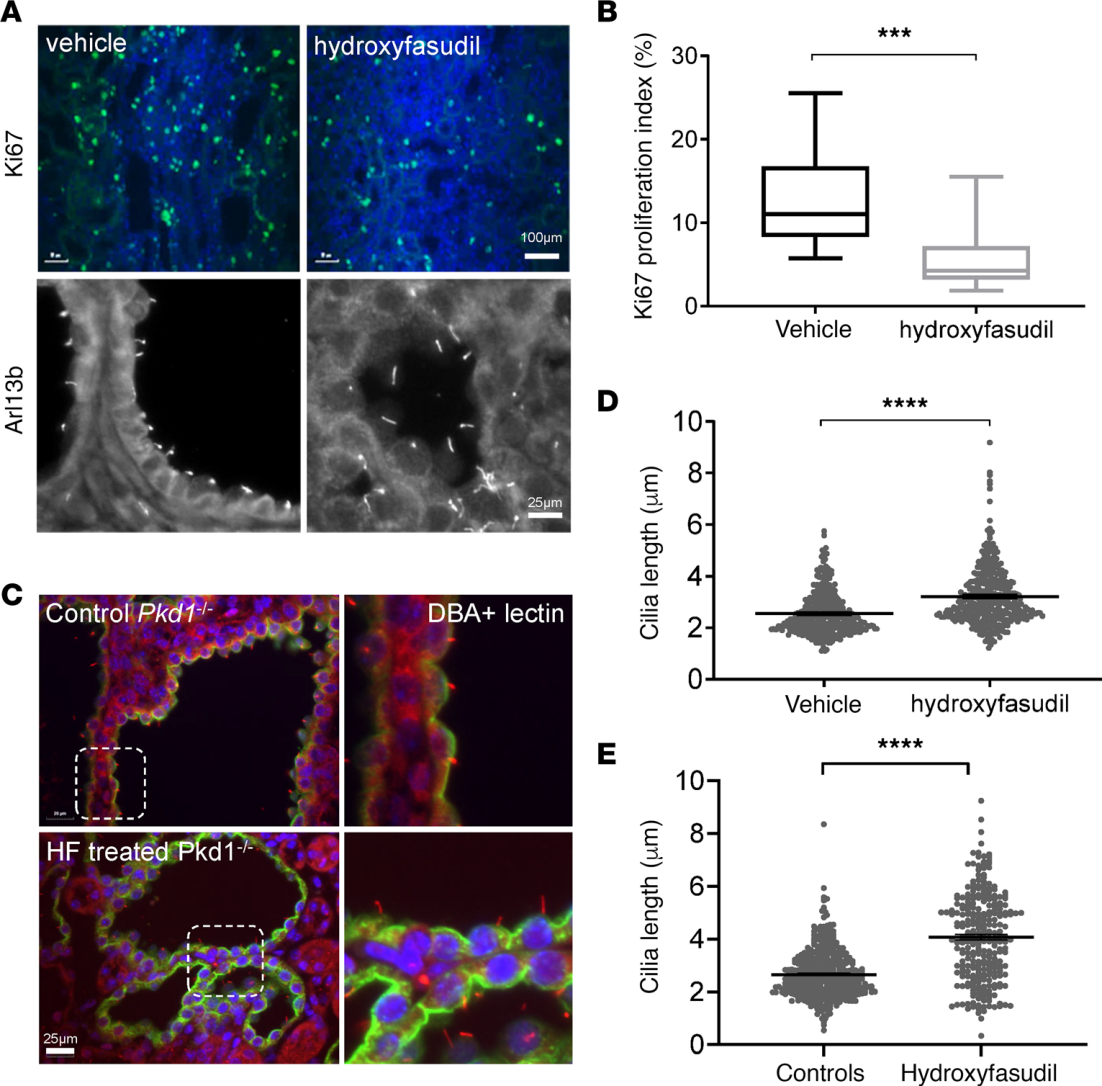
Changes in cell proliferation and cilia length in *Pkd1* mice after hydroxyfasudil treatment. (**A**–**D**) The proliferation index (Ki67) was significantly reduced (**A** and **B**) and cilia length (Arl13b) significantly increased (**C** and **D**) in hydroxyfasudil-treated animals (50 cilia per animal). (**E**) Representative images of primary cilia in *Pkd1*^–/–^ kidney tissue. DBA lectin–positive collecting duct cysts were stained green and primary cilia (Arl13b) was labeled red. Dotted lined boxes show the region under higher magnification (original magnification, ×1000). Mean cilia length was significantly increased in DBA-positive cysts after hydroxyfasudil treatment (*n* = 3 independent experiments, *N* = 276 cells). ***P < 0.001, ****P < 0.0001. Significance determined by 2-tailed Student’s *t* test.

Hydroxyfasudil is the major metabolite of the fasudil, one of the first ROCK inhibitors, and has been shown to be effective in other preclinical models of kidney disease such as ischemia/reperfusion injury, diabetic nephropathy, and unilateral ureteric obstruction (21–23). We next tested the effectiveness of hydroxyfasudil in a tetracycline-inducible kidney-specific *Pkd1* mouse model (*Pax8^rtTA^-TetO-Cre-Pkd1^fl/fl^*) (24). Treatment with hydroxyfasudil (10 mg/kg/day) from PN16-PN22 after kidney-specific *Pkd1* deletion was well tolerated, as reflected by changes in daily body weights (Figure 6B). After 7 days, the treated mice had reduced fractional kidney weights (2KW/BW) and fractional kidney cystic indices compared with vehicle-treated controls (Figure 6, C–E). Consistent with these changes, we observed a significant reduction in the proliferative index (Ki67-positive cells) and an increase in cilia length in treated animals (Figure 7, A–D). The increase in cilia length seen in hydroxyfasudil-treated animals was also measurable when analysis was restricted to collecting duct-derived (DBA-positive) cysts suggesting that the observed changes in cilia length after treatment were unlikely to be secondary to treatment-induced changes in the origin of individual cysts or a segment-specific effect (Figure 7E). In this model, few cysts were derived from proximal tubules as shown by the lack of LTA staining (Supplemental Figure 4A). There was a nonsignificant decrease in BUN between vehicle- and hydroxyfasudil-treated *Pkd1* animals after 7 days treatment; however, at this stage of disease, there was just a small increase in BUN between uninduced and induced *Pkd1* mice. Hydroxyfasudil did not alter BUN in the control (uninduced) animals (Supplemental Figure 4B).

## Discussion

In this study, we report that dramatic changes in the actin cytoskeleton are a striking feature of the ADPKD cystic cellular phenotype. An unexpected finding was of reduced ciliation, in both the number of ciliated cells and the cilia length in *PKD1* cystic cells, in *PKD1* and *Pkd1* cystic kidney tissue. To exclude the possibility of confounding factors such as genetic background, secondary genetic or epigenetic changes related to prolonged passage or cell immortalization, we generated isogenic *PKD1*-null cells by CRISPR/Cas9 mutagenesis and found similar changes, thus implicating this directly to PC1 expression. The molecular basis for this phenotype appears to be an increase in centrosome RhoA activation, leading to elevated ROCK activity and increased F-actin polymerization and contractility. Using cilia length as a phenotypic readout for centrosomal RhoA activity, we identified 3 candidate ARHGAP proteins (ARHGAP5, -29, -35) as essential regulators of normal ciliation, whose centrosome location had been reported but whose function had not been previously defined. Nonetheless, we found that PC1 expression was essential only for the centrosomal localization of ARHGAP35. We therefore conclude that the centrosome retention of ARHGAP5 and -29 are determined by their interaction with other proteins apart from PC1. A recent paper reported a glomerulocystic phenotype in an *Arhgap35* (p190A RhoGAP) mutant mouse model generated in an ENU mutagenesis screen (25). The amino acid substitution (p.Leu1396Gln) leads to loss of function, resulting in increased RhoA activity and reduced ciliogenesis (number and length) rescued by ROCK inhibition (25). Our findings confirm these results but extend them by providing the evidence of a mechanistic link between ARHGAP35 and PC1 in cyst formation. It should be noted that homozygous *Arhgap35* mutant mice developed hypoplastic kidneys and heterozygous mice had normal kidneys, with a low prevalence (<10%) of glomerular cysts. We conclude that a reduction in centrosomal PC1-ARHGAP35 interaction is likely to contribute to cyst formation in ADPKD but there could be compensation by other ARHGAPs such as ARHGAP5, -29, and others. Equally, other reported signaling pathways (centrosome and noncentrosome) likely contribute to cyst initiation and expansion in the ADPKD kidney. Although we have demonstrated that PC1 can bind to ARHGAP35, PC1 is mainly localized at the plasma and ciliary membrane while ARHGAP35 is localized to centrosome and noncentrosome (cell-cell, cell-matrix) compartments. It is plausible that both proteins could interact at the plasma membrane in noncentrosomal locations (see below). Nevertheless, since centrosomal ARHGAP35 localization and/or retention is clearly dependent on PC1 (Figure 4), we speculate that this could relate to the trafficking and delivery of ARHGAP35 with PC1 to the centrosomes in the same vesicles (26, 27) and/or its retention at the centrosomes with the cleaved PC1 C-terminus (CT1) (28) (Figure 6).

**Figure 8:**
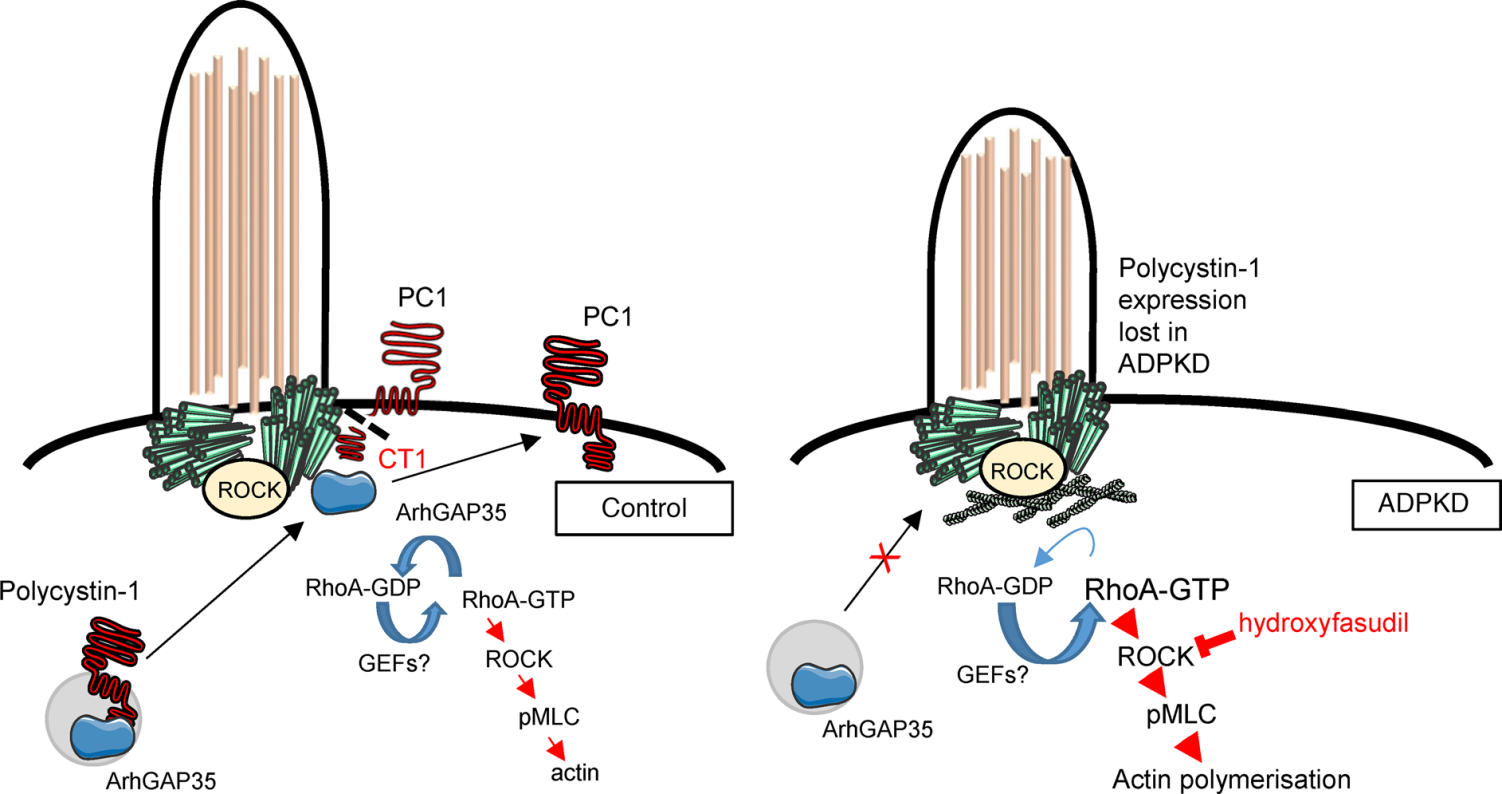
Mislocalization of centrosomal ARHGAP35 due to PC1 mutation leads to accumulation of active RhoA, ROCK activation, increased actin polymerization, and shorter cilia in ADPKD. A model showing how mutation of PC1 could lead to reduced cilia/centrosomal localization or retention of ARHGAP35. Two possible scenarios are shown: (a) trafficking and delivery of PC1 and ARHGAP35 in the same vesicles to the centrosome compartment and (b) retention of cleaved PC1 C-terminus (CT1) bound to centrosomal ARHGAP35. The RhoA-dependent kinase, ROCK, has been previously shown to be localized to centrosomes (32). Loss of centrosomal ARHGAP35 leads to the accumulation of centrosomal “active” GTP-RhoA, the activation of ROCK and its downstream effectors (e.g., pMLC), leading to increased actin polymerization and shorter cilia. It is plausible that the local increase in centrosomal ROCK activity could lead in turn to a cascade of ROCK activation, which spreads throughout the cell. PC1, polycystin-1; ADPKD, autosomal dominant polycystic kidney disease.

The increase in RhoA activation in *PKD1* cells is probably not restricted to the centrosome compartment as reflected by the observed increase in total cellular GST-RhoA and pMLC expression (Figure 3). The functional relevance of these findings to ADPKD pathogenesis was confirmed using the selective ROCK inhibitor hydroxyfasudil in vitro by 3D cyst assays using human-derived *PKD1* cystic cells and in vivo using a previously reported *Pkd1-*inducible mouse model. It seems likely that the beneficial effect of ROCK inhibition in these models extends beyond the centrosomal actin subcompartment; however, it is possible that this could be the initiating signal for local RhoA activation, which then spreads throughout the cell (Figure 8).

During the course of this study, 2 other groups reported the beneficial effects of 2 other ROCK inhibitors in 2 different neonatal *Pkd1* mouse models, lending support to our findings (29, 30). In both studies, a functional link between increased RhoA/ROCK activity as a relevant upstream regulator of YAP/TAZ in ADPKD was reported. Nonetheless, our finding of a molecular link between PC1 and centrosomal ARHGAP35 is since neither study provided a direct mechanistic link to PC1. Our findings also imply that this is a very proximal or early change in cystic pathogenesis due to the molecular link between ARHGAP35 and PC1. Taken together, RhoA appears to be a common upstream molecule whose activity is altered by several abnormalities seen in ADPKD, i.e., changes in cell-cell, cell-matrix, and centrosome-cilia interactions.

Our results confirm the likely significance of the centrosome compartment both for cilia formation and as a major signaling node for regulating cellular function. Recent papers have shown that the centrosome is not only the main cellular microtubule-organizing center but also an actin filament-organizing center (31). The descriptions of several other centrosomal ARHGAPs and ARHGEFs from proteomic studies and a previous report of centrosomal ROCK localization argue for the importance of fine-tuning compartmentalized RhoA/ROCK activity and consequent local actin dynamics both in health and disease (16, 32). The functional importance of each regulator, how they interact in local complexes, and their relevance in ADPKD are important areas for future study. It is also plausible that PC1 may regulate the actin cytoskeleton in other cellular compartments where it has been localized (cell-cell junctions and cell-matrix contacts) (33, 34) through the recruitment or stabilization of other ARHGAPs and/or ARHGEFs. In this context, it should be noted that ARHGAP35 has also been localized to both focal adhesions and cell-cell junctions in other cell types (35, 36).

Cdc42 has been reported to promote cilia formation through the localization of the exocyst complex to the cilia base and kidney-specific cdc42 deletion is associated with cyst formation (37). Although we detected an increase in active Cdc42, expression of dominant-negative Cdc42 did not alter cilia length in our *Pkd1* cystic cells. These could reflect cell type or species differences. Nonetheless, Cdc42 activation could contribute to noncentrosomal changes in actin and microtubular dynamics in our cellular models, relevant to the cystic phenotype (38).

Centrosomal PC2 localization has been reported in several studies; its function in this compartment is presently unclear. Notably, PC2 has been shown to bind to several centrosomal proteins, including pericentrin and SCLT1, and may localize to the cilia base through binding the exosome protein Sec10/EXOC5 (37, 39, 40). It is also notable that PC2 has been shown to bind several actin-binding proteins (mDia1, actinin-4, filamin-A), which have been reported to regulate its channel activity (41–43). Nonetheless, in our study, PC2-null cells had normal cilia length, suggesting that the cilia phenotype is primarily related to changes in PC1 expression.

Our findings of reduced cilia length in the absence or mutation of PC1 are consistent with previous studies in both primary and immortalized *PKD1* cystic cells (44, 45). The current literature, however, has reports of normal, reduced, or longer cilia length in association with PC1 deficiency. The first study of cilia length in *Pkd1^del34^* collecting duct-derived embryonic kidney cells (E15.5) reported “well developed” cilia lengths (although formal measurements were not provided) in the context of loss of flow-induced, cilia-mediated signaling (46). However, a later report using the same cells reported shorter cilia (with occasional long cilia) and associated centrosomal abnormalities: in this study, increased expression of SIRT2 was causally linked to these changes (47). Conversely, *Pkd1*-transgenic mice develop longer renal cilia in noncystic tubules (48), although, curiously, this phenotype has also been observed in precystic and/or cystic tissues and cells derived from several *Pkd1*-deficient mice, i.e., the *Pkd1^RC/RC^*-hypomorphic mouse (49), *Pkd1-* and *Pkd2-*null embryonic (E15.5) kidney epithelial cells (50), and *Arl13b*-transgenic *Pkd1* mice (51). Because changes in PC1 dosage have been associated with variable changes in cilia length (none, reduced, increased) in different model systems, we conclude that this is not an essential feature of disease. The regulation of cilia length is complex and likely to be determined by multiple factors influencing both actin and microtubule-dependent mechanisms in disease (52). Differences between species, genetic background, segmental origin (44), differentiation, and proliferative status (embryonic vs. adult onset) (53), the presence of other functional cilia transgenes (e.g., *Arl13b*) (51, 54), organ involvement (kidney vs. liver) (49), inflammation (55), recovery from injury (56), senescence (57), mechanical forces (flow, stretch), or physical constraints (cell shape in 3D tissues) (58) could all be relevant modifying factors. A more systematic study examining these factors in other disease models will be needed. Alterations in cilia signaling, however, could be the common abnormality in all these models regardless of cilia length, although there are currently opposing views on whether cilia themselves exert a negative effect on cell proliferation acting as a “brake” on the cell cycle (59) or a positive effect through an unidentified “cilia-dependent cyst-activating signal” (24) in the context of ADPKD.

ROCK inhibitors have been clinically approved for use in the treatment of glaucoma and vasospasm, although their wider applications have been limited so far by systemic side effects (60). Our results indicate that dysregulation of the RhoA/ROCK axis in ADPKD is likely to be a major factor in cyst initiation and should stimulate the development of further therapeutic approaches in this area.

## Methods

### Materials.

All chemicals were purchased from Sigma Chemical (Poole), unless otherwise stated. Plasmids were obtained through Addgene as indicated. The following antibodies were used in this study: PC1 (7e12), PC2 (g20), actin, ARHGAP5, ARHGAP29, myc, streptavidin, GST (Santa Cruz Biotechnology), ArhGAP35, MLC and pMLC (Cell Signaling), Flag (Sigma), RhoA, Cdc42 and Rac1 (Cytoskeleton), and Arl13b (Proteintech).

### Cell Lines.

Noncystic (UCL93, CL5, CL8, CL11) and cystic (OX161, OX938, SKI001, SKI002) human kidney epithelial cells were generated and cultured as previously described (61–63). Cilia formation was induced by serum starvation for 48 hours at 37°C.

### CrispR/Cas9 mutagenesis.

UCL93 cells were transfected with pSpCas9(BB)-2A-Puro (PX459) V2.0 [pSpCas9n(BB)-2A-Puro (PX462) V2.0; gift of Feng Zhang, Broad Institute, Cambridge, Massachusetts, USA; Addgene plasmid 6298] (64) containing a gDNA targeting the first exon of *PKD1* (5′-CACCGCGCCGGGCGCTGGGCCGCAG) or the first exon of *PKD2* (5′-CACCGCGTGGAGCCGCGATAACCC). Positive clones were selected for puromycin resistance followed by limiting dilution. Mutations were then validated by genomic DNA sequencing and Western blotting using specific antibodies to PC1 (7e12) and PC2 (1A11) (65, 66).

### Transfections.

Cells were transfected using Lipofectamine 3000 (Life Technologies) for 48 hours before the cell assays. For siRNA knockdown assays, cells were transfected with negative control or specific ARHGAP siRNAs (SmartPool) using RNAimax (Life Technologies). siRNA knockdown was confirmed by qPCR using specific TaqMan probes.

### Western blotting and IP.

Total cell lysates were prepared and processed for IP and Western blotting as previously described (67). Cells were solubilized in detergent lysis buffer (50 mM Tris, 0.14 M NaCl, 1% Triton X-100, and 0.5% NP40) supplemented with cOmplete Protease Inhibitors and PhosStop Phosphatase Inhibitors (Roche Diagnostics). IP and GST pulldown assays were performed as previously described (26); ECL detection and quantification were performed using a Bio-Rad ChemiDoc XRS+ system running Image Lab automated image capture and analysis software. All quantification was carried out on nonsaturated bands as determined by the software from 3 independent experiments.

### BioID proximity assay.

A common centrosomal targeting domain identified from pericentrin and AKAP450 (PACT) (68) or the C-terminal domain of PC1 (CT1) was cloned into myc-BioID2-MCS (myc-BioID2-MCS; gift of Kyle Roux, Sanford Children’s Health Research Center, San Diego, California, USA; Addgene plasmid 74223) (69). After transfection, cells were incubated with 50 μM biotin (B4501, Sigma) overnight. Lysates were prepared as described and incubated with 50 μl Dynabeads MyOne Streptavidin C1 (Thermo Fisher Scientific) for 4 hours. The beads were washed 6 times with lysis buffer, bound proteins eluted, and separated by SDS-PAGE before analysis by immunoblotting with specific ARHGAP primary antibodies.

### Active RhoA, Cdc42, and Rac1 pulldown assays.

Levels of GTP-bound RhoA were determined using a Rhotekin-RBD bead pulldown assay. Levels of GTP bound Cdc42 or Rac1 were determined by PAK-PBD beads pulldown assays (Cytoskeleton) as described. GTP-γ– and GDP-treated cell lysates were used as positive and negative controls, respectively. Samples were separated by SDS-PAGE and analyzed by immunoblotting with RhoA, Cdc42, or Rac1 antibodies.

### Rho-GTPase biosensor.

The active RhoA biosensor GFP-rGBD (gift of William Bement, University of Wisconsin–Madison, Madison, Wisconsin, USA; Addgene plasmid 26732) (12) was transfected into UCL93 or OX161 cells. After serum starvation, to induce cilia formation, the proportion of cells localizing GFP-rGBD at the cilia base was quantified.

### Polycystin rescue experiment.

UCL93 and OX161 cells were transfected using Amaxa electroporation (Lonza) (program W-01) with CFP-PC2 (27) and mCherry-PC1 or mCherry-PC1-4211X (gifts of Peter Harris, Mayo Clinic, Rochester, Minnesota, USA (70). After transfection, cells were serum starved for 48 hours at 37°C to induce cilia formation. Cilia were stained with Arl13b and measurements were performed on mCherry-PC1–transfected cells with an Olympus Imaging Systems inverted IX-71 microscope set to capture cellular fluorescence images with a CCD camera (Hamamatsu), driven by Simple PCI software (C Imaging Systems).

### Rapamycin-induced centrosome translocation.

YF-RhoA (CA) (Addgene plasmid 20153) and Lyn11-targeted FRB (LDR) (Addgene plasmid 20147) were gifts of Tobias Meyer, Stanford University, Stanford, California, USA (71). The PACT centrosomal targeting domain was cloned into LDR replacing the Lyn11 plasma membrane targeting domain. UCL93 cells were transfected with YF-RhoA (CA) and PACT-FRB-HA. Translocation of YF-RhoA to the centrosome was induced 24 hours later by the addition of 10 μm rapamycin (Calbiochem) for 10 minutes and washed, followed by serum starvation for 48 hours at 37°C to induce cilia formation. Cilia were stained with Arl13b and measurements were performed on an Olympus Imaging Systems inverted IX-71 microscope set to capture cellular fluorescence images with a CCD camera (Hamamatsu), driven by Simple PCI software (C Imaging Systems).

### Immunofluorescence staining.

Immunofluorescence staining was performed as previously described (26, 27). Primary cilia were visualized using an antibody to Arl13b (Proteintech). HA- and myc epitope–tagged proteins were detected with anti-HA and anti-myc polyclonal antibodies (Santa Cruz Biotechnology) and Alexa Fluor 488 or 594 secondary antibodies (Invitrogen). F-actin was detected using Rhodamine-Phalloidin (Invitrogen). For mouse tissue, serial sections were dewaxed and rehydrated and antigen retrieval was carried out using Tris-EDTA (pH 9). DBA- and LTA lectin–positive cysts were identified using FITC-conjugated lectins (Vector Labs). Slides were viewed using an Imaging Systems inverted IX71 microscope (Olympus) configured for multifluorescence image capture. Images were acquired using SimplePCI imaging software (Compix). For cilia length measurements, NIH ImageJ analysis software was used to measure greater than 100 cilia in at least 3 independent experiments. Cytochalasin D and the ROCK inhibitor Y-27632 were added to cells at the indicated concentrations for 3 hours before cilia length measurement. Super resolution microscopy was carried out on a DeltaVision/GE OMX optical microscope (version 4) for structured illumination (3D-SIM) and analyzed using Imaris image analysis software (Bitplane).

### Matrigel 3D cyst assays.

3D Matrigel cyst assays were performed as previously described (62). In brief, OX161/C1 cells (1 × 10^5^/well) were mixed with 70 μl Matrigel (Becton Dickinson), plated into 96-well plates in triplicate, and incubated for 30 minutes at 37°C to facilitate gel formation. Cells were then cultured for 12 days in the presence of hydroxyfasudil (Tocris). Media was replaced every 2 days. The average cyst area was calculated by measuring cyst areas in individual wells on day 12. At least 65 cysts were measured in triplicate wells at each time point.

### Effect of hydroxyfasudil in vivo.

*Pkd1* deletion was induced by doxycycline injections at postnatal days (PNs) 13–15 in tetracycline-inducible, kidney-specific *Pkd1* mice (*Pax8^rtTA^-TetO-Cre-Pkd1^fl/fl^*) (72). Experimental animals were injected i.p. with hydroxyfasudil (10 mg/kg/day) or vehicle (sterile water) for 7 days from PN16. After sacrifice, following a schedule 1 method, blood and tissues were rapidly collected. Blood was collected and centrifuged at 2000 *g* for 10 minutes to collect serum that was quickly snap-frozen in liquid nitrogen and stored at –80°C until further analysis. Each kidney was cut transversally into 4 pieces. The top and bottom sections were snap frozen for biochemical analysis, whereas the middle section was embedded in cry-M-bed solution (Wolflabs) or immersed in 10% Neutral Buffered Formalin (MilliporeSigma) for histological analysis. After termination at PN23, terminal 2KW/BW was calculated and tissue sections were analyzed for cystic index, cilia length (Arl13b), and proliferation index (Ki67). Serum blood urea nitrogen measurements were carried out by the Department of Clinical Chemistry at Sheffield Children’s Hospital Foundation Trust.

### Statistics.

Data are presented as mean ± SEM. Two-tailed Student’s *t* test and one-way ANOVA corrected for multiple comparisons were used for statistical analysis, with *P* values greater than 0.05 considered statistical significance.

### Study approval.

Animal studies were approved by the University of Sheffield Medical School and carried out under Home Office license PF2A3AD69. A tetracycline-inducible, kidney-specific *Pkd1* mouse model (*Pax8^rtTA^-TetO-Cre-Pkd1^fl/fl^*) on a C57/BL6 background was a gift from the late David Huso (Baltimore PKD Center, Baltimore, Maryland, USA).

## Author contributions

PPP conducted experiments, acquired data, and analyzed data. AJS conducted experiments, designed research studies, acquired and analyzed data, and helped to write the manuscript. AO obtained funding, designed research studies, analyzed data, and wrote the manuscript.

## Supplementary Material

Supplemental data

Supplemental Video 1

Supplemental Video 2

## Version 1 07/14/2020

In-Press Preview

## Version 2 08/20/2020

Electronic publication
