# Supplementary figures and images for: Polycystin-1 regulates ARHGAP35-dependent centrosomal RhoA activation and ROCK signaling
Source: JCI Insight. 2020 Aug 20;5(16):e135385. doi: 10.1172/jci.insight.135385 (PMC7455122; doi:10.1172/jci.insight.135385)

Supplementary Figure 1

A

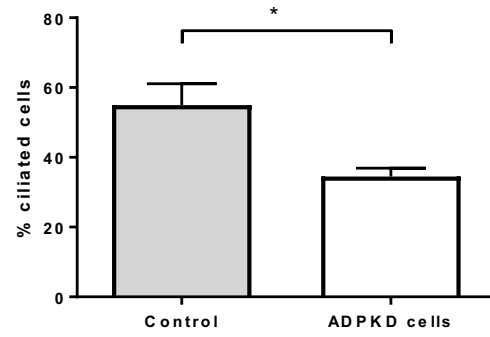

B

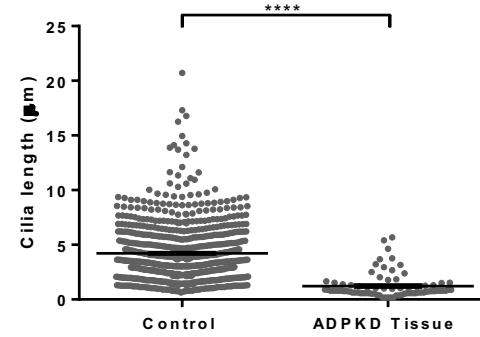

C

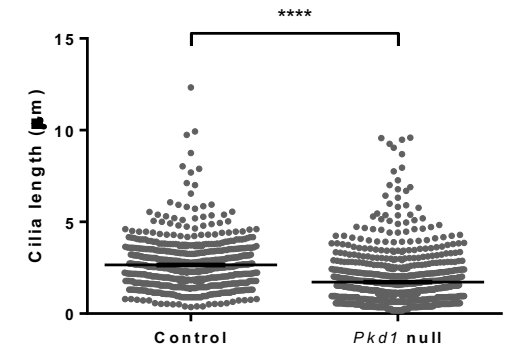

D

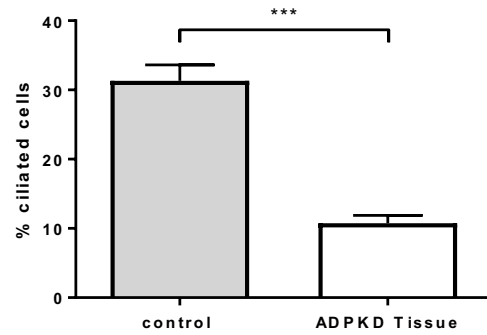

E

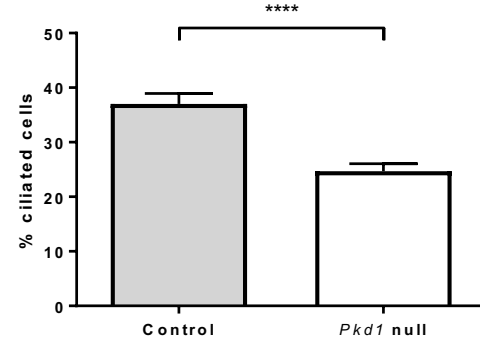

F

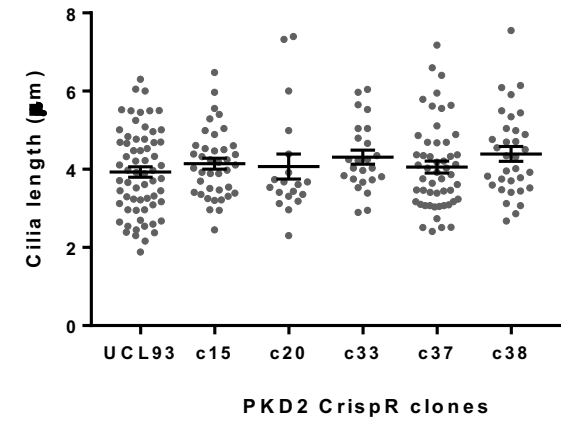

A

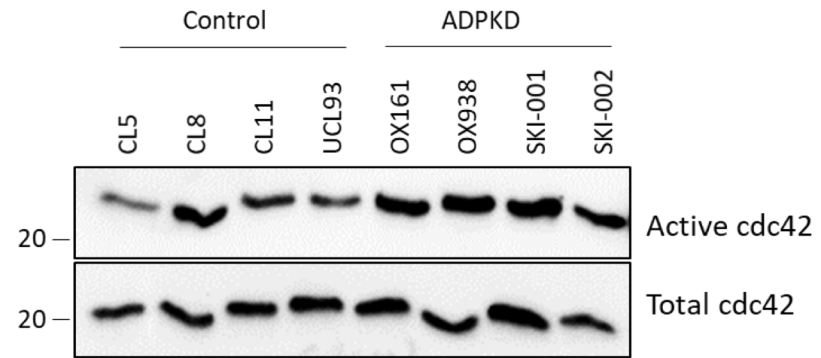

B

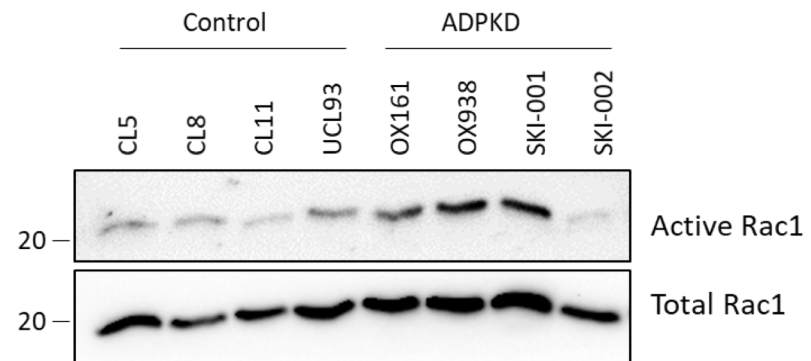

C

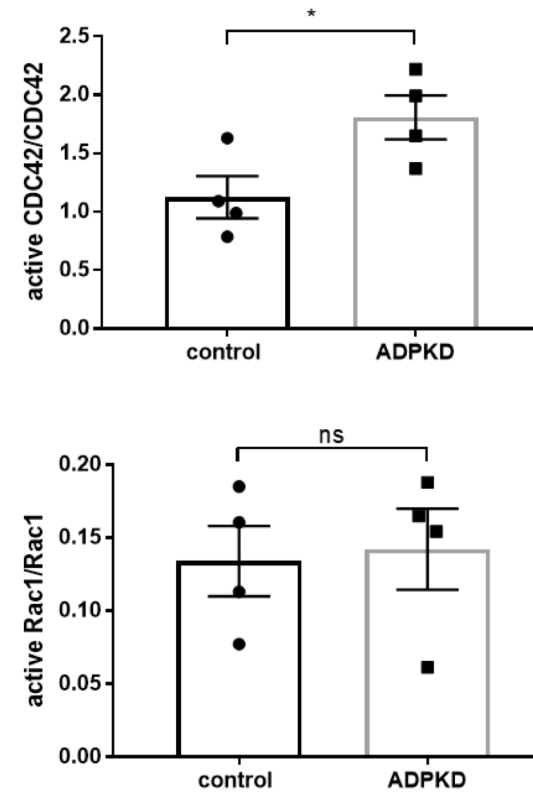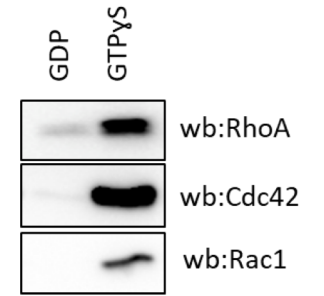

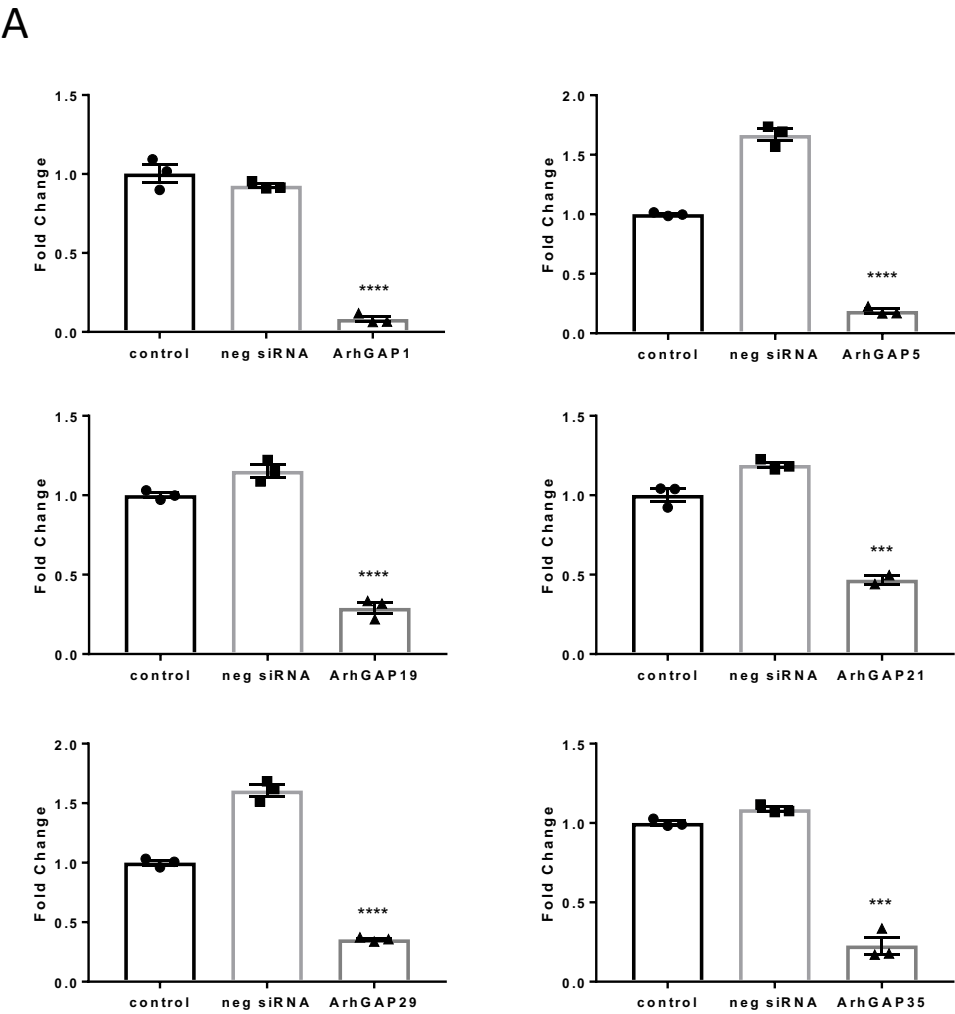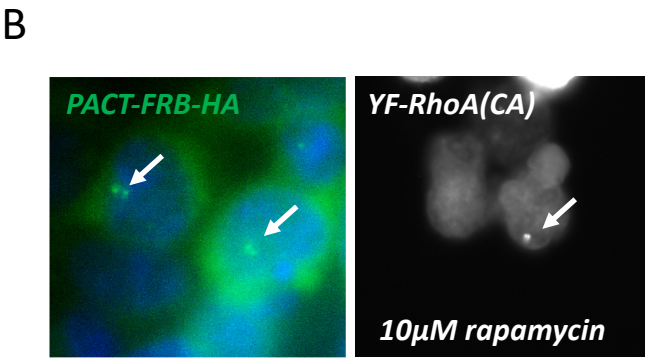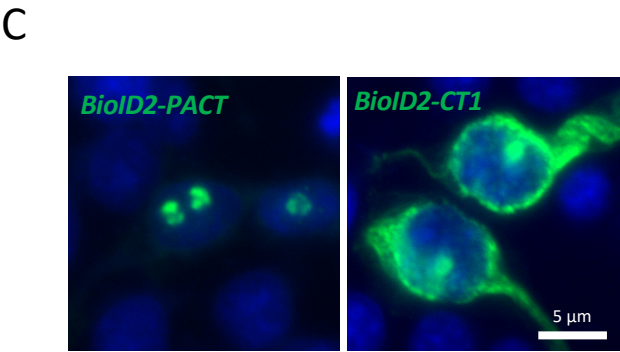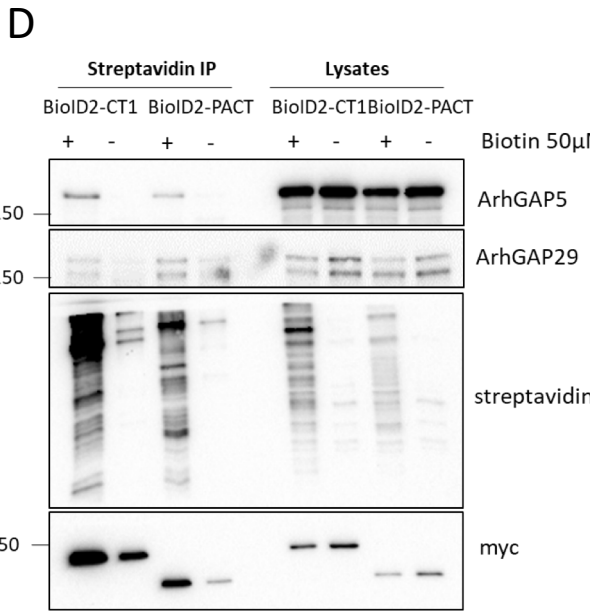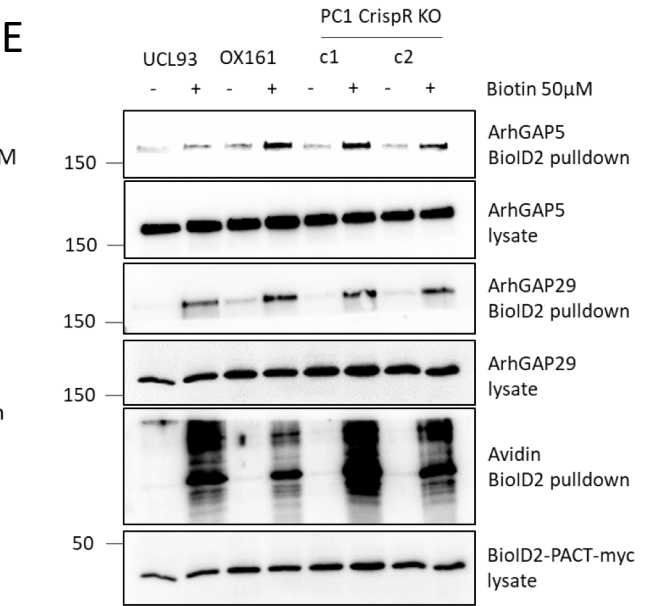

A

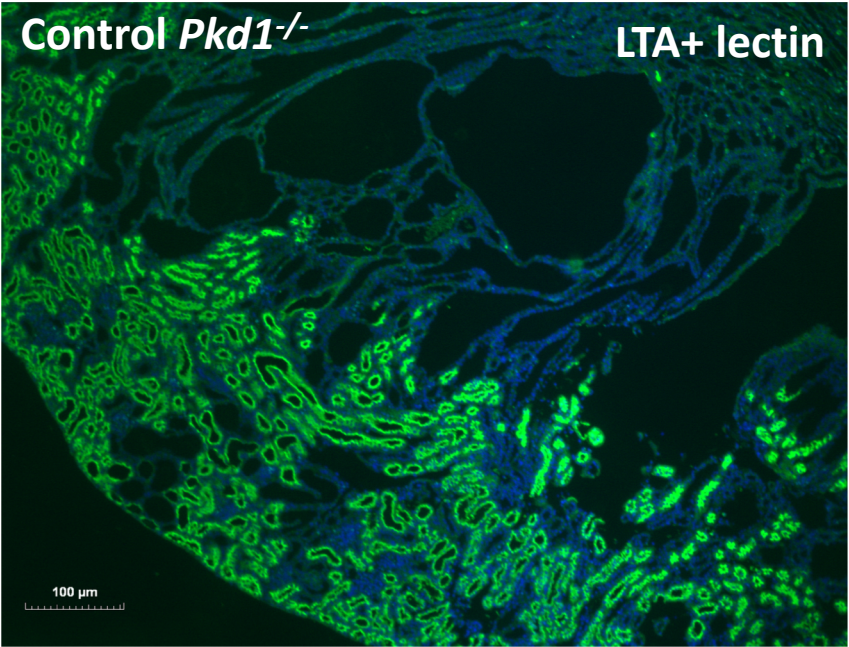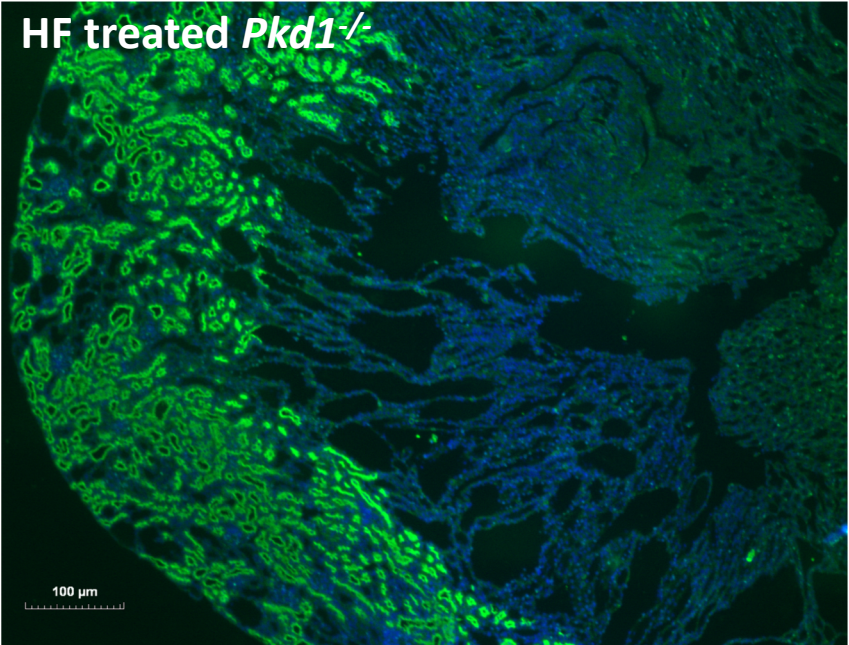

B

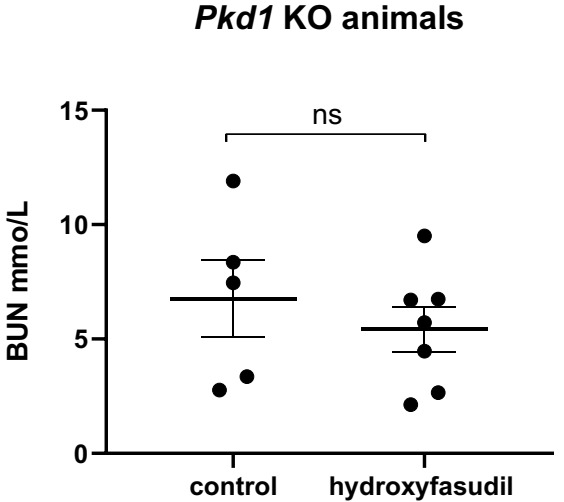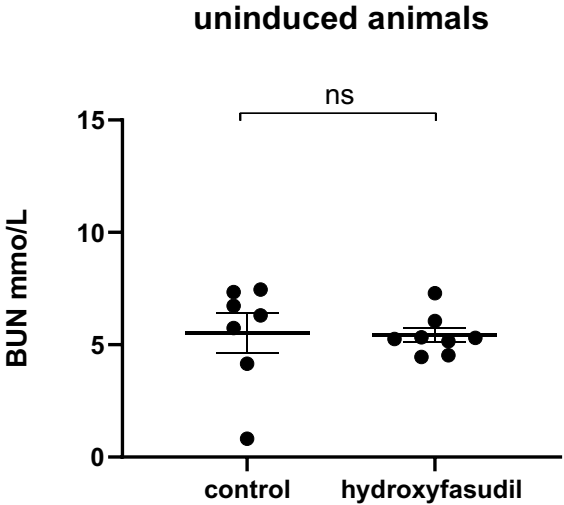

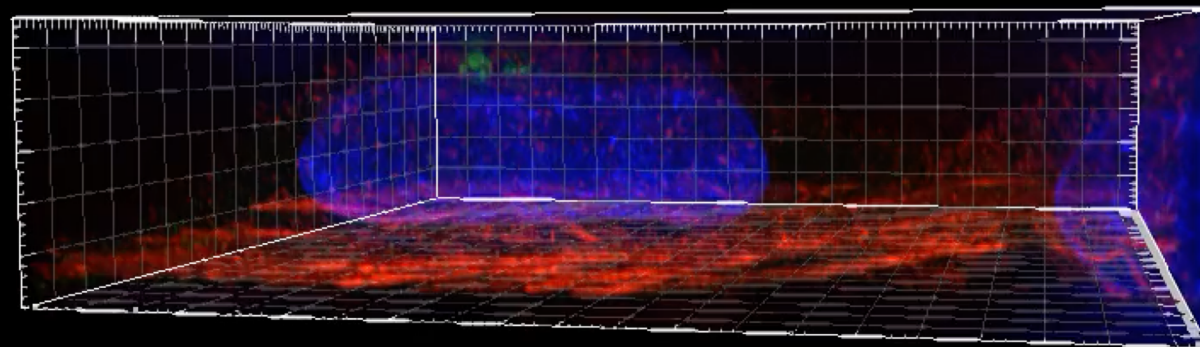

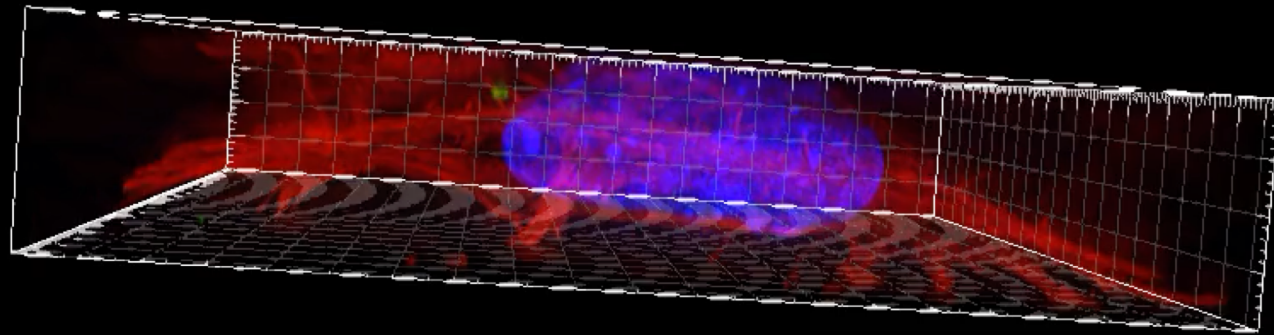

Supplement: Supplemental data [file jciinsight-5-135385-s160.pdf]
